# Prevalence of Human Immunodeficiency Virus and associated factors among Visceral Leishmaniasis infected patients in Northwest Ethiopia: a facility based cross-sectional study

**DOI:** 10.1186/s12879-017-2261-8

**Published:** 2017-02-17

**Authors:** Mekuriaw Alemayehu, Mamo Wubshet, Nebiyu Mesfin, Abebaw Gebayehu

**Affiliations:** 1 Institute of Public Health, College of Medicine and Health Sciences, University of Gondar, Gondar, Ethiopia; 2 Department of Public Health, St. Paul’s Hospital Millennium Medical College, Addis Ababa, Ethiopia; 3 School of Medicine, College of Medicine and Health Sciences, University of Gondar, Gondar, Ethiopia

**Keywords:** Visceral Leishmaniasis, Human immunodeficiency virus, Coinfection, Northwest Ethiopia

## Abstract

**Background:**

Visceral Leishmaniasis coinfection with HIV/AIDS has emerged as a series of disease pattern. It most often results in unfavorable responses to treatment, frequent relapses, and deaths. Scarce data is available regarding the prevalence of HIV and associated factors among Visceral Leishmaniasis coinfected patients. This study sought to determine the prevalence of HIV and associated factors among Visceral Leishmaniasis infected patients.

**Methods:**

Facility based cross-sectional study was conducted from October, 2015 to August, 2016 in Northwest Ethiopia. Cluster sampling technique was used to select 462 Visceral Leishmaniasis infected patients. Serologic and parasitological test results have been used to diagnose Visceral Leishmaniasis. The HIV diagnosis was based on the national algorithm with two serial positive rapid test results. In case of discrepancy between the two tests, Uni-Gold ^TM^ was used as a tie breaker. Structured questionnaire was used to collect independent variables. Data was entered by using Excel and analyzed by using SPSS version 20. Descriptive statistics and logistic regression model was used to analyze the data.

**Results:**

A total of 462 study participants were included in the study with a response rate of 92.4%. HIV and Visceral Leishmaniasis coinfection was found to be 17.75% with 95% CI; 14.30–21.40. Age ≥ 30 years (AOR = 22.58, 95% CI 11.34, 45.01), urban residents (AOR = 2.02, 95% CI 1.16, 4.17) and daily laborer workers (AOR = 4.99, 95% CI 2.33, 10.68) were significantly associated with HIV and Visceral Leishmaniasis coinfection.

**Conclusion:**

HIV and Visceral Leishmaniasis coinfection in the Northwest Ethiopia was found to be low. Age, residence and employment were independently associated with HIV-VL coinfection in the Northwest Ethiopia. It is better to design interventions to prevent and control HIV-VL coinfection for productive age groups (age ≥ 30) and daily laborers.

## Background

Visceral Leishmaniasis (VL; also known as “kala-azar”) is a systemic parasitic disease caused by the parasite *Leishmania donovani* species complex. It is estimated about 500,000 new cases of VL occur annually worldwide [1]. VL is characterized by irregular bouts of fever, substantial weight loss, swelling of the spleen and liver, and anemia (which may be serious). If the disease is not treated, the fatality rate in developing countries can be as high as 100% within 2 years [2]. VL accelerates HIV replication and disease progression, mainly by chronic immune stimulation [3].

The prevalence of patients with both HIV and VL infection (hereafter, “HIV-VL coinfection”) in Europe has fallen sharply since 1996, when antiretroviral treatment (ART) became standard [4, 5]. In India and particularly in Africa, HIV-VL coinfection is emerging [4, 5]. The AIDS pandemic has expanded to rural areas where VL is endemic, with cases of HIV-VL coinfection reported in 35 countries [4, 5], among which Ethiopia carries the greatest burden. The affected populations are mainly very poor male seasonal migrant workers that travel in the harvesting season from non endemic highlands to the cotton, sesame and sorghum fields of Humara and Metama, the VL endemic low lands situated on the Sudanese boarders [6, 7].

In Ethiopia, HIV prevalence has declined from 1.5% in 2011 to 1.1% in 2015 [8]. Hence, in spite of the decreasing prevalence of HIV in the general population, the prevalence of HIV among VL patients has remained proportionally very high. The prevalence of HIV-VL coinfection from different studies in Ethiopia range from 18.1 to 48.5% [9, 10]. The real burden is likely to be underestimated or overestimated because of rapid decrease of HIV infection in Ethiopia [8]. There is however knowledge gap on the current prevalence of HIV among VL infected patients.

In most of the studies done outside Ethiopia, factors associated with HIV-VL coinfection were advanced HIV-1 disease [11, 12], intravenous drug users [11, 13], CDC clinical category C [14, 15] and CD4 cell count below 300 cells/mm^3^ [15]. Nevertheless, one hospital based case series study done in Ethiopia showed that age was significantly associated with HIV-VL coinfection [16]. Therefore, there is a scarcity of data on factors associated with HIV-VL coinfected patients in Ethiopian context.

This study is aimed to determine the prevalence of HIV and associated factors among VL infected patients in the endemic areas of Northwest Ethiopia. The findings of this study could be useful evidence for scholars who are interested in the field and the ART programs undertaken by the government and non – government organizations.

## Methods

### Study design

Facility based cross-sectional study design was employed to assess the prevalence of HIV and associated factors among VL patients who visited the health facilities in Northwest Ethiopia.

### Study settings and population

From the VL treatment centers found in the Northwest Ethiopia, three hospitals and one health center were selected purposely considering the availability of invasive VL diagnostic methods such as demonstration of parasite from spleen/lymph node aspiration or positive serology test if the patient has no VL history. In addition to VL diagnostic method, we also considered the availability of Fluorescence Activate Cell Sorting (FACS) count machine for CD4 count and CBC (complete blood count) machine. Hospitals and Health centers found in the study area that fulfilled the above considerations were considered as clusters (units). The selected Hospitals and Health center are the only health facilities that have well organized VL diagnosis and treatment centers found in the study area. The excluded health facilities in our study have not yet started diagnosing and treating VL patients. If VL patients visited these health facilities then they will be referred to one of the selected health facilities.

The study was carried out at four different sites in Northwest Ethiopia. The first site was Abdrafi inpatient kala-azar treatment center located in Abdrafi; at this health center medical services are provided for patients with Leishmaniasis, HIV-VL coinfection and snake bite. The second site was kala-azar treatment and research center in the University of Gondar Hospital located in Gondar; at this center both outpatient and inpatient medical services are provided for patients with Leishmaniasis and HIV-VL coinfection in addition to the comprehensive medical service from other units of the University of Gondar Hospital. The third site was Kahsay Aberra Hospital located in Humera kala-azar treatment center; at this center both outpatient and inpatient medical services are provided for patients with Leishmaniasis, HIV-VL coinfection and many other hospital level services. The fourth site was Metema Hospital located in Metema kala-azar treatment center; at this center both outpatient and inpatient medical services are provided for patients with Leishmaniasis, HIV-VL coinfection and many other hospital level services.

The sample size (n) was computed by single population proportion formula *n* = [(zα/2) ^2^ × P (1-P)]/d^2^ by assuming 95% confidence level of Z_α/2_ = 1.96, margin of error 5% and we have taken proportion of 18.1% HIV-VL coinfection conducted in endemic area of Amahara region [10]. By considering this; the calculated sample size was 226.8 with adjustments for design effect of 2 and for non response rate (10%) the final sample size became 500. The included study participants were 155, 79, 89 and 139 patients at Abdrafi health center, Metema hospital, Humera hospital and University of Gondar hospital respectively.

The study population was all VL patients who visited VL treatment facilities found in Northwest Ethiopia. Cluster sampling technique was employed to include study participants. Therefore, Abdrafi Health center, Metema Hospital, Humera Hospital, and University of Gondar Hospital were the four selected clusters. All VL diagnosed patients who visited and admitted to these facilities were included in the study. In addition to this, we have included HIV-VL coinfected patients who already started receiving ART and VL patients who are already started VL treatment during the beginning of the study. All the included study participants were admitted to the selected VL treatment centers and their admission was because of their VL infections and not for other disease. Participants who were mentally incompetent and unable to speak to undertook consent and interviews were excluded from the study. The study period was from October 7/2015 to August 5/2016.

### Measurements

Diagnosis of VL was conducted according to the guidelines for the diagnosis of Leishmaniasis in Ethiopia [17]. The WHO case definition of VL was used as a starting point; history of fever for more than 2 weeks, malaria excluded, in combination with wasting and either splenomegaly or lymphadenophaty [18]. A patient whose illness met this case definition and who had no previous VL treatment was diagnosed serologically by positive rK39 rapid diagnostic test (Diamed-IT-Leish, DiaMed AG) [19]. Patients with previous VL history underwent splenic or lymph node aspiration and VL confirmed parasitologically. A severely ill patient with a negative rK39 test was aspirated without delay, so that a diagnosis could be made as quickly as possible.

The selected health facilities have got both parasitological and serological (rk39 dipstick test) VL diagnosis methods. The standard means of parasitological diagnosis in VL entails microscopy and/or culture from spleen, bone marrow or lymph node. While highly accurate, the procedure is invasive, painful, and carries the risk of potentially fatal bleeding. In order to avoid such problems and taking into account the patient have no previous VL infection we used serological tests for 46.32% of the included study participants. But if the patient has previous history of VL infection then the serological tests were less effective. Hence, we have used parasitological tests for 53.68% of the included study participants. VL diagnosis was made by laboratory technologists working in the selected health facilities. After spleen or bone marrow aspirations were made as appropriate, laboratory technologists and/or senior clinicians read the aspirates at least twice. Wright or Giemsa staining was used as available in the health facilities.

Provider-initiated testing and counseling for HIV was offered to all VL patients. The HIV diagnosis was based on the national algorithm with two serial positive rapid test results; The KHB (Shanghi Kehua Bio-engineering, ltd, 2008, China) HIV test was used to diagnose HIV. For positive results, confirmation were done using STAT-PAK test (chembio diagnostic system Inc, 2008, USA). In case of discrepancy between the two tests, Uni-Gold™ (Trinity Biotech PLC, Bray, Ireland) was used as a tie breaker. As VL is considered a stage IV-defining illness in HIV patients [18, 20], all patients were given ART as soon as they were stabilized from their acute illnesses. ART regimens follow the national guidelines: tenofovir-lamivudine-efavirenz; zidovudine-lamivudine-efavirenz; or zidovudine-lamivudine-nevirapine [21] Second-line ART consists of protease inhibitor-based combination regimens.

The clinical and treatment related data’s were extracted from the chart of each patient by using checklist. Data on demographic factors were collected by using structured and pretested questionnaire which was developed by the investigators. The structured questionnaire was prepared in English version and translated into Amharic (local language) and again back to English to confirm the correctness of the translation. The data collectors were 4 nurses and 4 laboratory technologist. We employed four physicians and health officers as the supervisors of the data collectors. One day training was given to the data collectors and supervisors on the data collection tool and sampling techniques. Supervision was held regularly during data collection period. The collected data was reviewed and checked for completeness and relevance in each day before going to the next day data collection.

### Definition of variables

The dependent variable was HIV-VL coinfection and independent variables were socio-demographic variables, clinical characteristics and treatment related variables. The variables were defined as categorical variables with the following:

- ***HIV***-***VL coinfection*** – *A person who was positive for both VL and HIV diagnosis*.
- ***Spleen size*** - *Spleen size of 15 cm and above is defined as a huge splenomegaly*, *and is associated with infarction*, *anemia and dragging abdominal pain*.
- ***Altitude adjusted hemoglobin***: − *the adjustment is subtracted from each individual*’*s observed hemoglobin level to calculate adjusted hemoglobin. The altitude of Gondar from sea level which is found to be 2*,*133 m which will be adjusted by subtracting 0.8 g*/*dl from the observed hemoglobin. The other places have an altitude of less than 1000 m and there is no adjustment*. [22].

### Data analysis

Each completed questionnaire for socio-demographic, clinical and treatment related variables was checked visually for completeness before fed to the computer. The data was entered into Excel, data cleanup and cross-checking was done and it was analyzed by using SPSS version 20. Descriptive statistics like frequencies and cross tabulation was performed. All variables with *P*-value <0.2 in bivariate analysis were included into a multivariate step wise backward logistic regression model. Goodness of fit for model was checked by Hosmer and Lemeshow test. Hence, the assumption fitted the test at *P*-value = 0.78 > 0.05. Crude and adjusted odds ratios with 95% confidence interval was used to determine the strength of association between dependent and independent variables. Variables having *P*-value ≤ 0.05 was considered as significant.

## Results

A total of 462 study participants were included in the study with response rate of 92.4%. The mean age (±SD) of the included study participants was 26.47 (±9.19) years. Ninety six point five percent (96.5%) of the study participants were males. Majority of the respondents 95.24% were Orthodox Christians and 4.55% were Muslim in religion. Almost half of the study participants (49.80%) were unable to read and write. Three hundred thirteen (67.75%) were reported single. Two hundred twenty one (47.84%) of the included study participants were farmers. Among the study participants 230 (49.78%) were urban residents (Table 1).

**Table 1:** Socio-demographic, clinical and treatment related characteristics of the study participants in Northwest Ethiopia, 2016

Variables	Frequency	Percent
Sex		
Male	446	96.50
Female	16	3.50
Age (years), mean (±SD) = 26.47 (±9.19)		
≤ 10	8	1.73
11–20	121	26.19
21–30	223	48.27
31–40	71	15.37
41–50	28	6.06
≥ 51	11	2.38
Religion		
Orthodox Christian	440	95.24
Muslim	21	4.55
Protestant	1	0.22
Ethnicity		
Amhara	315	68.33
Tigray	125	27.11
Kimant	10	2.17
Oromo	5	1.08
Others	6	1.30
Residence		
Urban	230	49.78
Rural	232	50.22
Education level		
Not read and write	230	49.80
Read and write	51	11.00
First cycle (1–8) & above	181	39.20
Marital status		
Single	313	67.75
Married	97	21.00
Divorced	52	11.26
Employment		
Farmer	221	47.84
Daily laborer	138	29.87
Government employed	8	1.73
Non government employed	15	3.25
Merchant	74	16.02
Housewife	6	1.29
CD4+ count (mg/dl), mean (114.41)		
< 100	314	67.97
≥ 100	148	32.03
Observed Hb, mean (±SD) = 8.54 (±2.15)		
< 8.54	225	48.7
≥ 8.54	231	50.0
Adjusted Hb, mean (±SD) = 8.29 (±2.14)		
< 8.29	220	47.6
≥ 8.29	236	51.1
Spleen size, mean (±SD) = 7.66 (±4.88)		
< 15	346	74.9
≥ 15	35	7.6
Duration of ART for HIV-VL patients		
Not started	32	39.02
< 12 *months*	11	13.41
12–36 months	19	23.17
≥ 37	20	24.39
Duration of VL treatment		
≤ 5 days	172	37.23
6–15 days	98	21.21
≥ 16 days	192	41.56

Eighty two (17.75 with 95% CI; 14.30–21.40) of the included study participants were found to be HIV-VL coinfected patients. Among the HIV-VL coinfected patients 54 (65.85%) were found to have a CD4 count less than 100 cells/mm^3^. During diagnosis of VL the mean (± SD) observed hemoglobin was 8.54 mg/dl (±2.15) and 216 (46.75%) of the study participants had hemoglobin less than 8.5 mg/dl. After we adjusted for the altitude, the mean (± SD) altitude adjusted hemoglobin was 8.29 mg/dl (±2.14) and 226 (48.92%) of the study participants had hemoglobin less than 8.29 mg/dl. During diagnosis of VL 346 (74.89%) of the study participants had a spleen size less than 15 cm (Table 1). Thirty two (39.02%) of the coinfected patients were newly diagnosed for HIV-VL concurrent infection. Hence, they were not started antiretroviral treatment (Table 1).

In the bivariate analysis residence, marital status, employment and age of the participant were significantly associated with HIV-VL coinfection (Table 2).

**Table 2:** Bivariate associations of the levels of HIV-VL coinfection with socio-demographic and clinical factors among VL infected patients in Northwest Ethiopia, 2016

Characteristics of the study participants	HIV-VL Coinfection		COR (95% CI)	*P*-value
	Yes	No		
Sex				0.579
Male	80	366	1.53 (0.34, 6.87)	
Female	2	14	*r*	
Age				0.001
< 30	19	303	*r*	
≥ 30	63	77	13.05 (7.37, 23.09)	
Residence				0.001
Urban	60	170	3.37 (1.99, 5.72)	
Rural	22	210	*r*	
Marital status				0.001
Single	35	278	*r*	
Married	20	77	2.06 (1.13, 3.78)	
Divorced	27	25	8.58 (4.49, 16.39)	
Employment				0.010
Farmer	28	193	*r*	
Daily laborer	35	103	2.34 (1.35, 4.07)	
Others^a^	19	84	1.56 (0.83, 3.95)	
Education				0.124
Not read and write	41	189	1.24 (0.73, 2.10)	
Read and write	14	37	2.16 (1.03, 4.52)	
First cycle and above	27	154	*r*	
Observed Hb				0.385
< 8.54	43	182	1.24 (0.76, 2.01)	
≥ 8.54	37	194	*r*	
Altitude adjusted Hb				0.554
< 8.29	41	179	1.16 (0.71, 1.88)	
≥ 8.29	39	197	*r*	
Spleen size (cm)				0.304
< 15	64	282	*r*	
≥ 15	4	31	0.57 (0.19, 1.67)	
CD4+ count (mg/dl)				0.651
< 100	54	260	*r*	
≥ 100	28	120	1.12 (0.66, 1.92)	

*r* reference, ^a^government employed, non government employed, housewife and merchant, the stated *P*-values are the overall *P*-values. Pearson Chi-square test was used to generate the *P*-values

The multivariate analysis was used to identify factors that were predictive of HIV-VL coinfection. Age, residence and employment were independently associated with HIV-VL coinfection (Table 3).

**Table 3:** Multivariate association of the levels of HIV-VL coinfection with factors among VL infected patients in Northwest Ethiopia, 2016

Variables	HIV-VL coinfection		COR (95% CI)	AOR (95% CI)	*P*-value
	Yes	No			
Age					
< 30	19	303	*r*	*r*	*P* = 0.001
≥ 30	63	77	13.05 (7.37, 23.09)	22.58 (11.34, 45.01)	
Residence					
Urban	60	170	3.37 (1.99, 5.72)	2.20 (1.16, 4.17)	*P* = 0.016
Rural	22	210	*r*	*r*	
Marital status					
Single	35	278	*r*		
Married	20	77	2.06 (1.13, 3.78)		
Divorced	27	25	8.58 (4.49, 16.39)		
Employment					
Farmer	28	193	*r*	*r*	*P* = 001
Daily laborer	35	103	2.34 (1.35, 4.07)	4.99 (2.33, 10.68)	
Others^a^	19	84	1.56 (0.83, 3.95)	3.74 (1.57, 8.93)	
Educational status					
Not read and write	41	189	1.24 (0.73, 2.10)		
Read and write	14	37	2.16 (1.03, 4.52)		
First cycle & above	27	154	*r*		

*r* reference, ^a^government employed, non government employed, housewife and merchant; the stated *P*-values are the overall *P*-values

## Discussions

This study focused on determining the prevalence of HIV and associated factors among VL infected patients. As a result, the prevalence was 17.75% and factors such as age, residence and employment were associated with HIV-VL coinfection. This is in line with other studies which reported socio-demographic factors might have an effect on HIV-VL coinfection [5, 10]. In addition to this, there are few emerging articles which recommend detailed studies on HIV-VL coinfection [23, 24].

As to this study result, the prevalence of HIV-VL coinfection was found to be 17.75%. This finding is almost equal with the study conducted in Amhara region and Humera Northwest Ethiopia which showed that the proportion of HIV-VL coinfection was 18.1 and 18.6% respectively [10, 25]. But this finding is lower than other studies done in Ethiopia by using similar method, Army Hospital, Addis Ababa from 1992 to 2001; Humera, Northwest Ethiopia from 1998 to 2000; Humera, Northwest Ethiopia, 2004; Gondar University Hospital, Northwest Ethiopia from 1999 to 2004; Gondar University and Humera Hospital, Northwest Ethiopia, from 2006 to 2008 in which HIV-VL coinfection was 48.5, 23, 28.5, 41 and 38.2% respectively [16, 26–29]. This might be due to the effectiveness of national strategy on interventions of behavioral change to reduce vulnerability to HIV infection designed by government and non government organizations. The other reason might be due to raised awareness on using interventions to prevent VL infection such as using insecticide treated bed net to prevent sandfly bites.

Age of the study participants was found to be significantly associated with HIV-VL coinfection. The study participants with age ≥ 30 years had 22.58 times risk for HIV-VL coinfection than the participants with age < 30 years. This was in line with the study done in Gondar University Hospital which showed that VL patients of age group > 20 years were more than 3 times risk to have HIV infection as compared to those 20 years and below [16]. The age group distribution in coinfected patients is slightly higher in our study but the bottom line is HIV-VL coinfection mainly strikes adults. This study also gives additional evidence on the HIV-VL coinfection mainly risks the productive age groups (age ≥ 30) and this will help to identify the target group for intervention.

Employment of the participants was positively associated with HIV-VL coinfection in which those who were daily laborers had 4.99 times risk for HIV-VL coinfection than farmers. In addition to this, other professionals such as government employees, non government employees, housewives and merchants together had 3.74 times risk for HIV-VL coinfection as compared to farmers. The possible explanation for the first one is daily laborers were mobile seasonal/migrant workers who came from highlands to VL endemic lowland areas for economical reason. Therefore, daily laborers are likely to have no pre-existing immunity to VL and/or lack of awareness about VL prevention methods as compared to farmers who were resided in the lowlands. The possible explanation of VL coinfection for the later is farmers were benefitted from the massive scale-up of insecticide treated nets for malaria control launched by Ministry of Health in 2005 that may therefore have collateral benefit for VL control [30]. The other reason might be due to effectiveness of health extension workers on interventions of behavioral change to reduce vulnerability of HIV and VL infection of farmers [31].

Residence of the study participants was found to be associated with HIV-VL coinfection. Those who were from urban residence had 2.2 times risk for HIV-VL coinfection as compared to rural residents. This finding is in line with the current national HIV/AIDS progress report of Ethiopia such as urban residents are more affected by HIV than rural residents [8]. The reason behind the VL coinfection might be those urban residents had a travel history to endemic areas for different reasons such as to work as a daily laborer or for other reason like trading purpose. Therefore, they might have no pre-existing immunity to VL and/or lack of awareness of VL prevention mechanism as compared to farmers who permanently resided in low land areas.

In our study, CD4 cell count, Hb, and spleen size of included patients did not significantly associated with HIV-VL coinfection but this may be because of the small sample size of the study and majority of the study participants were male (97%). This is therefore makes unlikely to obtain a significant association of the HIV prevalence in this population since females are twice affected than male population with HIV in Ethiopia [8]. Indeed, one study has shown that CD4 cell count is important predictor of HIV-VL coinfection [15].

The findings of this study should be interpreted with some limitations. The study relies on participants who manage to come to the health institutions. We might not get HIV-VL coinfected patients who couldn’t visit the health facilities for different reason. Therefore, the estimated prevalence may not exactly show the HIV-VL coinfection burden in the community. Moreover, self report of historical VL events of the study participants were used during VL diagnosis. Hence, recall bias could have present. Majority of the study participants were male sex and a little younger age groups; these may introduce some bias to our study. The reason for not including more female sex and older age groups were HIV-VL coinfected female and older age group patients were not found at all the health centers during the study period.

## Conclusion

HIV-VL coinfection in the Northwest Ethiopia was found to be low. Age, residence and employment were independently associated with HIV-VL coinfection in the Northwest Ethiopia. It is better to design interventions to prevent and control HIV-VL coinfection for productive age groups (age ≥ 30) and daily laborers.

## Abbreviations

ART: Antiretroviral therapy
ARV: Antiretroviral drug
CBC: Complete blood count
CD4: Cluster of differentiation 4
FACS: Fluorescence Activate Cell Sorting
rK39: Recombinant K 39
SPSS: Statistical package for social science
VL: Visceral Leishmaniasis
WHO: World Health Organization
